# Induction of T Cell Senescence by Cytokine Induced Bystander Activation

**DOI:** 10.3389/fragi.2021.714239

**Published:** 2021-07-09

**Authors:** Attiya A. Abbas, Arne N. Akbar

**Affiliations:** Division of Medicine, University College London, London, United Kingdom

**Keywords:** ageing, immunosenecence, bystander activation of T cells, senescent T cells, NK-like CD8(+) T cells

## Abstract

As people around the world continue to live longer, maintaining a good quality of life is of increasing importance. The COVID-19 pandemic revealed that the elderly are disproportionally vulnerable to infectious diseases and Immunosenescence plays a critical role in that. An ageing immune system influences the conventional activity of T cells which are at the forefront of eliminating harmful foreign antigens. With ageing, unconventional end-stage T cells, that exhibit a senescent phenotype, amass. These senescent T cells deviate from T cell receptor (TCR) signaling toward natural killer (NK) activity. The transition toward innate immune cell function from these adaptor T cells impacts antigen specificity, contributing to increased susceptibility of infection in the elderly. The mechanism by which senescent T cells arise remains largely unclear however in this review we investigate the part that bystander activation plays in driving the change in function of T cells with age. Cytokine-induced bystander activation may offer a plausible explanation for the induction of NK-like activity and senescence in T cells. Further understanding of these specific NK-like senescent T cells allows us to identify the benefits and detriments of these cells in health and disease which can be utilized or regulated, respectively. This review discusses the dynamic of senescent T cells in adopting NK-like T cells and the implications that has in an infectious disease context, predominately in the elderly.

## Introduction

Until recently, T cells were considered to only differentiate towards an end-stage through repeated life-long antigen activation, for example, by persistent viruses such as cytomegalovirus (CMV) (Vasto et al., 2007; Fulop et al., 2013; Tu and Rao, 2016). These end-stage T cells, that exhibit characteristics of senescence, accumulate during ageing and have been shown to exhibit low proliferative activity after activation and were therefore considered to be dysfunctional (Deursen, 2014). It was assumed therefore, that age-associated decline of immunity or immune senescence, was attributed in part to the development of deficient T cell function over time. However instead of being dysfunctional, senescent-like T cells, especially within the CD8 compartment, lose key components of their T cell receptor (TCR) signalling apparatus while concomitantly acquiring Natural Killer (NK cell) characteristics (Pereira et al., 2020). This indicates that as T cells differentiate towards a proliferative end-stage, they acquire a new functional perspective that is independent of their antigen specificity. Therefore, T cell function is re-focussed rather than lost during ageing and in this review, we discuss that antigen-independent bystander activation of T cells by cytokines may drive senescence as well as expression of NK-like activity in T cells. We also explore potential beneficial and immune-pathogenic roles for these cells in health and disease, especially those that induce intense inflammatory responses.

## The Phenotypic and Functional Characteristics of Senescent T Cells

Although end-stage T cells exhibit many characteristics of senescence, they are not senescent by classical definition as they can be re-induced to proliferate by blocking key signalling pathways (Goronzy and Weyand Nat Rev. Immunol. 2020). Nevertheless, in this review we refer to end-stage T cells as senescent T cells for simplicity. Senescent T cells in both the CD4 and CD8 compartments can be identified and isolated by the loss of cell surface expression of co- stimulatory molecules such as CD27 and CD28 while acquiring the expression of KLRG1 and CD57 (Covre et al., 2020). In addition, these cells have short telomeres, low telomerase activity, low proliferative capacity and express senescence related molecules including Atm, γH2AX, the cyclin inhibitor p16, sestrins, and p38 MAP kinase (Pereira et al., 2020). These senescent T cells are also characterised by their high levels of expression of senescence-associated β-galactosidase (SA-βGal) activity that can be measured by flow-cytometry (Martinez-Zamudio et al., 2021). Senescent T cells can also re-express CD45RA and despite the loss of proliferative capacity, these cells have potent cytotoxic activity and secrete pro-inflammatory cytokines such as TNFα and IFNγ after activation (Akbar et al., 2016; Pereira et al., 2020).

## Senescent T Cells Accumulate During Ageing and in Inflammatory Diseases

Senescent T cells increase during ageing, due in part to responses to lifelong infection with viruses such as cytomegalovirus (CMV) and Epstein-Barr virus (EBV) (Nikolich-Žugich J, 2018). An abundance of senescent T cells that have pro-inflammatory potential are found in age-related diseases including rheumatoid arthritis (Goronzy et al., 2013; weyand et al., 2017), Alzheimer’s Disease (Panossian et al., 2002; Franco et al., 2006; Gate et al., 2020), and cardiovascular diseases (Yu et al., 2015; Youn et al., 2019). Senescent T cells correlate with lesion size in cutaneous leishmaniasis (CL), a disease characterized by intense inflammatory responses and the formation of destructive cutaneous lesions (World Health Organization/Department of control of neglected tropical diseases, 2015; Covre et al., 2019). During acute infection with *L. braziliensis* senescent CD4 and CD8 T cells home to the skin by upregulating the skin homing receptor CLA (Covre et al., 2019). There, these senescent T cells can initially control the infection. However, the potent inflammatory activity of senescent T cells also contributes to the development of skin pathology in CL, even after parasites have been cleared (Covre et al., 2020). Senescent T cells can also infiltrate and accumulate in the kidneys of patients with Systemic Lupus Erythematosus (Tahir et al., 2015). It is possible therefore, that the inflammatory potential of senescent T cells may contribute to the immunopathology that is associated with inflammatory diseases (Covre et al., 2020). As senescent T cells may also associate with inflammatory diseases, understanding how these populations are generated and how their functional activity is controlled is vital to clarify how these cells may switch from being beneficial to becoming detrimental for health.

## Senescent T Cells Acquire Characteristics of Natural Killer Cells

Optimal T cell activation requires co-stimulatory signals *via* CD28 and CD27 and the loss of these cell surface receptors by senescent T cells suggests their decreased ability for T cell activation (Pereira et al., 2020). In addition, senescent CD4^+^ T cells lose expression of key components of the TCR signalosome such as LCK, LAT, and phospholipase C gamma (PLC- γ) (Lanna et al., 2014; Lanna et al., 2017; Pereira et al., 2020) that underscores their hypo- responsiveness to TCR signaling. Despite this loss, these cells express elevated levels of cytotoxic granules and perforin and secrete abundant levels of pro-inflammatory cytokines after activation (Akbar et al., 2016). The senescence program in CD4^+^ and CD8^+^ T cells is regulated by stress sensor molecules known as the sestrins (Lanna et al., 2017; Pereira et al., 2020). Sestrin expression in T cells is induced by signalling processes that may arise from either DNA damage or nutrient deprivation (Lanna et al., 2017).

Interestingly, senescent but not non-senescent T cells that express sestrins also express both stimulatory and inhibitory NK receptors and acquire the ability to perform TCR independent functional responses (Pereira et al., 2020). The function of activating (NKG2D) and inhibitory (NKG2A) on senescent CD8^+^ T cells has been demonstrated previously (Pereira et al., 2020) and these cells can also express additional killer inhibitory receptors (KIR) but their function on senescent CD8^+^ T cells has not been investigated (Pereira et al., 2020). Sestrin inhibition restores TCR signalosome expression, proliferative and telomerase activity as well as re-inducing the expression of CD28 (Lanna et al., 2017; Pereira et al., 2020) while concomitantly decreasing both NKG and KIR receptor expression by these T cells (Pereira et al., 2020). Therefore, senescent T cells exhibit a reversible senescence program but can be induced to exhibit preferentially T cell or NK related functional activity by selective sestrin inhibition. The NK-like function of TEMRA cells is likely to be regulated by a balance of stimulatory and inhibitory NKR signalling.

The biological significance of the acquisition of NK receptors and functions by senescent T cells is unclear but there is an expansion of these cells in older individuals and especially in centenarians (Derhovanessian et al., 2013; Pawelec, 2014; Michel et al., 2016) suggesting that this may be a beneficial adaptation to ensure broad and rapid effector function during ageing. The potential beneficial and detrimental consequences of bystander activation of T cells have been discussed in excellent previous reviews (Kim and Shin, 2019). The reprogramming of senescent T cells from TCR to NKR functional activity could allow CD8^+^ T cells to specifically target persistent pathogens including CMV and the EBV and also malignant cells during ageing (Khan et al., 2004; Jackson et al., 2017; Pawelec et al., 2017).

## The Induction of Senescence Characteristics in T Cells by Cytokines

Surprisingly a literature survey suggests that a large proportion of senescent T cells may accumulate because of bystander activation by cytokines and not from antigen-specific stimulation *in vivo*. In this regard, type-1 interferons (IFN-1) plays a critical role in driving T cell senescence. Cytomegalovirus (CMV)-specific CD4^+^ T cells in healthy donors exhibit a greater extent of a senescent phenotype compared to other virus-specific T cells populations in the same individuals (Fletcher et al., 2005). This may be due in part to the ability of CMV to activate the secretion of large quantities of IFN-α by plasmacytoid dendritic cells, which inhibits telomerase activity and the loss of costimulatory molecules by activated CD4^+^ and CD8^+^ T cells *in vitro* (Fletcher et al., 2005; Lanna et al., 2013). Observations in hepatitis C virus-infected individuals on IFN-α therapy support this theory as they develop increased proportions of circulating CD8^+^ T cells with a CD28^−^ phenotype (Manfras et al., 2004). Interestingly, TNF-α is also induced at high concentrations *via* CMV stimulation and this cytokine can also downregulate co-stimulatory molecule, i.e., CD28 (Billadeau et al., 2003). IFN-α may also contribute to telomerase inhibition and telomere erosion in CD4^+^ T cells during recall antigen challenge in the skin *in vivo* (Reed et al., 2004). The possible mechanisms for inhibition of telomerase activity by this cytokine include the reduction of transcription and translation of human telomerase reverse transcriptase (hTERT) in stimulated CD8^+^ T cells (Lanna et al., 2013) and also the downregulation of NF-kB which drives hTERT transcription (Lanna et al., 2013). In other studies, inactivation of the IFN pathway has been found to extend the lifespan of *Terc* knockout mice indicating that DNA-damage response- induced IFN signalling as a key mechanism that links senescence and premature ageing (Yu et al., 2015). However, it is not clear at present if these cytokines regulate sestrin expression by senescent T cell populations.

Of note, IFN-1 signalling induces extensive T cell proliferation *in vivo* during infection with lymphocytic choriomeningitis virus (LCMV) (Tough et al., 1996; Dumont et al., 1986). Therefore, during viral infection, a proportion of T cell proliferation may be induced by bystander activation by IFN-1 (Buchmeier et al., 1980). Crucially, IFN-1 may not directly stimulate the proliferation (Zhang et al., 1998) but instead induce IL-15 that drives the proliferative activity in CD8^+^ T cells (Zhang et al., 1998; Tough et al., 2000). Memory CD8^+^ T cells may also be activated in a TCR—independent manner by other cytokines including IL-12 (Xiao et al., 2009), IL-18, or a combination of these (Lotze et al., 1981; West et al., 2011; Newby et al., 2017). In addition to the ability of IL-15 to induce proliferation after antigen stimulation *in vivo* (Kim and Shin, 2019), it can also induce NK activity in T cells (Kennedy et al., 1998; Castillo et al., 2012). This suggests that there may be interplay between cytokine-induced T cell senescence (through extensive proliferation) and NK receptor expression by these cells. Collectively these observations suggest that senescent T cells may also arise from bystander activation and not only from repeated antigen-specific T cell stimulation *in vivo*.

## The Induction of Natural Killer Receptors and function in T Cells by Cytokines

In 1985, Rosenberg and colleagues discovered that high-dose IL-2 incubation induces NK activity in CD8^+^ T cells, NK cells and invariant NK cells (Rosenberg et al., 1985; West et al., 2011). This was the beginning of the era of lymphokine (cytokine)-induced killer cells (CIK) (Fagan and Eddleston, 1987; Hartwing and Korner, 1990). More recently, CIK cells have been shown to be end-stage CD8^+^ T cells that are derived from a CD3^+^CD56^-^CD8^+^ T cell population (Franceschetti et al., 2009). These cells express polyclonal TCR Vβ chains and acquire the expression of CD56, NKG2D and exhibit granular lymphocyte morphology. The cytokine activated killer cells have a CD45RA^+^CCR7^-^ phenotype that is congruent with terminally differentiated (senescent) human memory T cells that re-express CD45RA (T_EMRA_) (Franceschetti et al., 2009). This supports the possibility that bystander activation of T cells may induce both senescence as well as NK receptor expression, but it is not clear if both outcomes are induced by the same mechanism.

IL-15 (but not IL-18) has been shown to confer NKG2D-mediated cytolytic function upon memory CD8^+^ T cells (Tough et al., 1999; Bastidas et al., 2014). NKG2D in CD8^+^ T cells can mediate costimulatory activity in the presence of TCR engagement (Groh et al., 2001: Jamieson et al., 2002; Roberts et al., 2001; Maasho et al., 2005). However, NKG2D signaling can also occur in the absence of TCR engagement (Lanier et al., 2015). In freshly isolated CD8^+^ TCRαβ^+^ intraepithelial lymphocytes (IELs) obtained from patients with active celiac diseases or IELs preactivated with IL-15, the cells displayed NKG2D-mediated cytotoxicity without TCR interaction (Roberts et al., 2001; Meresse et al., 2004). More recently, Hepatitis A virus (HAV)-infected cells obtained from acute hepatitis A (AHA) patients produced IL-15 which drove TCR-independent activation of memory CD8^+^ T cells via upregulation of NK receptors, NKG2D and NKp30 (Kim et al., 2018). IL-15 signalling stimulates the upregulation of NKG2D expression directly (Roberts et al., 2001; Meresse et al., 2004; Correia et al., 2011; Kim et al., 2018). However, NKG2D expression in bystander-activated CD8^+^ T cells is likely to be regulated through the coordinated interaction of many pro-inflammatory cytokines such as IFN-1 and IL-15, and TCR signalling (Kim and Shin, 2019).

IL-15 has also been found to drive the expression of NKG2A on mature T cells (Ponte et al., 1998; Mingari et al., 1998). NKG2A is a killer inhibitory receptor that commonly acts to block NK cell activity. However, NKG2A can also exert an inhibitory effect on TCR-mediated function on cytotoxic T cells. Therefore, the extent to which NKG2D or NKG2A is expressed by T cells or the differential expression of their ligands on target cells may potentially dictate whether there will be activation or inhibition of cytotoxicity.

## The Natural Killer Receptor Signalling Complex in Senescent CD8^+^ T Cells

An important question is how signalling pathways in senescent CD8^+^ T cells are re-wired to enable NK-like cytotoxic activity. Expression of NKG2D on the cell surface requires its association with adaptor proteins that stabilize the immunoreceptor complex and provide it with signalling activity (Franceshetti et al., 2009; Pereira et al., 2020). Immunoprecipitation studies showed that NKG2D associates with both the adaptor molecules DAP10 and DAP12. DAP10 contains a YxxM motif that activates PI3K leading to co-stimulation after TCR activation (Leong et al.: Gibbert et al., 2012), while DAP12 has an ITAM-motif that can recruit and activate Zap70-Syk to trigger cytokine release and cytotoxicity (Dunn et al., 2007; Zhu et al., 2008; Micco et al., 2013). Previous studies showed that in human CD8^+^ T cells, NKG2D is predominantly associated with DAP10 (Dunn et al., 2007; Zhu et al., 2008; Gibbert et al., 2012; Micco et al., 2013; Leong et al., 2014) which allows it to act as a costimulatory signal for the TCR. More recently, expression of DAP12 was found exclusively in senescent CD8^+^ T cells and was necessary and sufficient to mediate NKG2D- dependent cytotoxicity (Pereira et al., 2020). However, it is not known if DAP12 expression is also regulated by bystander cytokine activation.

Immunoprecipitation and imaging cytometry experiments indicated that DAP12, sestrin 2 and Jnk were co-localized in a complex with NKG2D in senescent but not in non-senescent CD8^+^ T cells (Pereira et al., 2020). Therefore, senescent T cells express a new signalling complex that include the sestrins that enable them to mediate NKG2D mediated cytotoxicity. However, the development of this new NK complex may be reversible since sestrin blockade can downregulate NKG2D and DAP12 but increase TCR related signalling molecules expression (Pereira et al., 2020). It will be important to determine the relationship between bystander activation, TCR activation and sestrin expression in senescent T cells. It remains to be determined how the sestrins interact with KIR receptors in senescent T cells.

## The Relationship Between Senescent T Cells and Virtual Memory T Cells

Virtual memory (VM) CD8^+^ T cells share certain characteristics with senescent CD8^+^ T cells. VM CD8^+^ T cells constitute up to 20% of the CD8 T cell population in murine lymphoid organs (Hogquist et al., 2014). These cells are generated by homeostatic mechanisms in response to self-antigens as well homeostatic cytokines, i.e., IL-7 and IL-15 (Hogquist et al., 2014). One of the key differences between VM cells and “true” memory T cells is that the former arise in germ free (GF) as well as “antigen free” mice, (Haluszczak et al., 2009), a situation that is unlikely to occur in humans. Comparably to senescent (TEMRA) CD8^+^ T cells (Callender et al., 2018), VM T cells have diminished proliferative potential in aged mice and humans, although they are highly proliferative in young individuals (Quinn et al., 2018). Interestingly, VM T cells accumulate during ageing and molecular analysis has revealed that aged VM T cells present a profile consistent with cellular senescence including reduced proliferative capacity, dysregulated MAPK signaling, and an elevated survival *in vitro* and *in vivo* with elevated expression of Bcl-2 (Quinn et al., 2018; Quinn et al., 2019). In mice, VM cells are characterized as CD44^high^CD122^high^CD49a^lo^ and EOMES positive (Haluszczak et al., 2009). Human VM T cells have not been well-defined, however like senescent T cells, they may arise as a result of bystander activation by cytokines (e.g. IL-15) (White et al., 2016) express NK receptors including NKG2A and KIR (Jacomet et al., 2015), accumulate with age, and show features of senescence (Quinn et al., 2018). This may indicate that VM T cells are a subset of or perhaps equivalent to the senescent CD8^+^ (TEMRA) cell population, especially in older individuals.

## Evidence for Bystander Activation of Heterologous T Cells During Viral Specific Immune Responses

**FIGURE 1:**
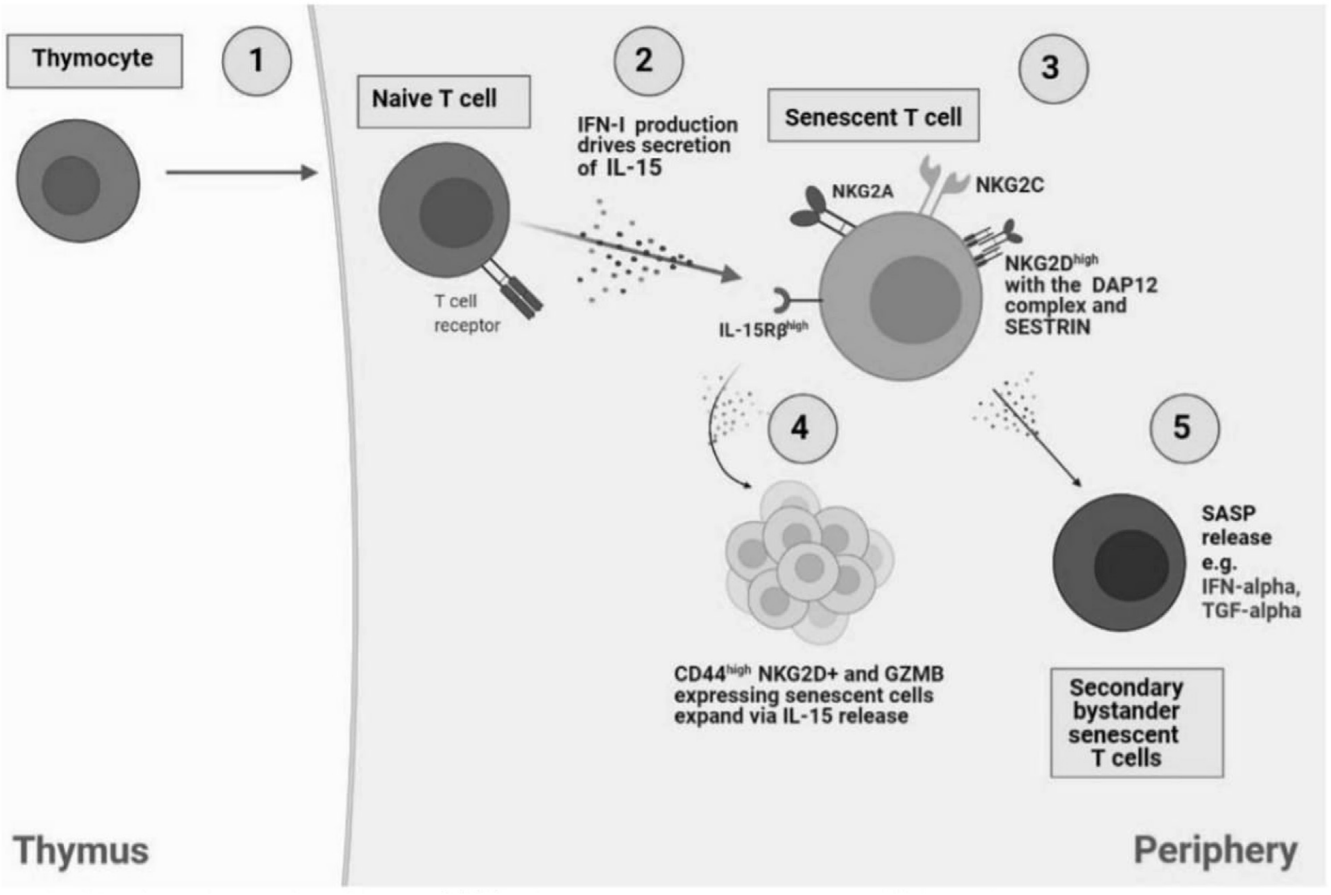
Bystander activation of NKR expressing senescent T cells. 1) In the thymus, IL-14 release from thymocytes drives cell differentiation into innate CD8 single positive thymocytes which migrate to the periphery to generate into memory T cells. However, naïve CD8+ T cells have the capacity to differentiate to senescent T cells in an antigen-independent manner, through cytokine stimulation, also termed bystander activation. 2) NaIve T cells produce IFN-1 which drives IL-15 secretion in neighbouring immune cells and the subsequent establishment of senescent T cells. 3) Senescent T cells express NK receptors including NKG2A, NKG2C, and notably NKG2D with the adaptor molecule, DAP12, driven by SESTRIN. 4) IL-15 secretion drives the expansion of NKG2D and CD44+ expressing senescent cells. 5) SASP release by senescent T cells, i.e., IFN-α and TGF-α can drive generation of secondary bystander senescent T cells.

These observations raise the possibility that there is bystander activation of T cells (Figure 1) by cytokines during specific immune responses to viruses *in vivo*. During acute hepatitis A virus (HAV) infection, IL-15 instead of the TCR activates CD8^+^ T cells that are specific for pathogens other than HAV itself, including human cytomegalovirus (HCMV), Epstein-Barr virus (EBV), influenza A virus respiratory syncytial virus, and vaccinia virus (Kim et al., 2018). As described above, the secretion of IL-15 could be secondary to the production of IFN-1 during the infection. The bystander activated CTLs in HAV infected individuals express high levels of cytotoxic molecules, i.e., NKG2D and GZMB (Kim et al., 2018). Bystander activation induced by IL-15 has also been indicated during human immunodeficiency virus (HIV) infection (Bastidas et al., 2014). The T cell repertoire of untreated HIV-infected patients showed that the TCR diversity of cycling effector memory CD8^+^ T cell reflects that of the complete effector memory CD8^+^ population and the activation of these cells is driven by non-specific activation (Younes et al., 2016). It has also been shown that bystander activation of CD8^+^ T cells occurs in the primary stages of HIV infection and activation markers, CD38 and HLA-DR, are upregulated in total CD8^+^ T cells and notably in the CD8^+^ T cells specific for HIV-unrelated viruses, i.e., HCMV, EBV, respiratory syncytial virus, and lymphadenopathy associated virus (LAV) (Dalod et al., 1999; Doisne et al., 2004; Kohlmeier et al., 2011). Other viral infections including Hepatitis B and influenza A also induce extensive bystander activation of T cells (Kim and Shin, 2019). This highlights the severity of antigen-independent activation of CD8 T cells in contributing to immunopathology. Therefore, it is important to further understand cytokine bystander activated T cells and how they may be regulated as a potential therapeutic to alleviate severe iimmunopathology during viral diseases.

## The Induction of Natural Killer Ligand Expression on Non-T Cells by Cytokines

While bystander cytokines can induce NKG2D expression in T cells, it begs the question of whether they can also induce the expression of NK ligands on different cell types. This could be a potential mechanism for non-specific tissue damage that leads to immunopathology. IFN-1 has been shown to induce the expression of MHC class I polypeptide–related sequence A (MICA) and UL16 binding protein (ULBP2) molecules that are MHC-related molecules and can both bind to and activate cells by NKG2D (Katlinskaya et al., 2015). Muscle biopsy specimens acquired from polymyositis (PM) patients show an upregulation of MICA/B correlating with IL-15 expression by muscle cells and the identification of CD8^+^ NKG2D^+^ cells within inflammatory infiltrates (Ruck et al., 2015). This suggests that NKG2D/IL-15 signaling pathway may induce muscle destruction in patients *via* CD8^+^ NKG2D^+^ cells targeting the MICA/B ligands that are upregulated by IL-15. Respiratory syncytial virus (RSV) can raise IL-15 production levels in respiratory epithelial cells *via* the NFkB pro-inflammatory pathway (Zdenrghea et al., 2012). This in turn leads to an upregulation of MICA on the surface of epithelial cells which can be eliminated *via* NK cell killing (Zdenrghea et al., 2012). In another study, IFN-y was shown to upregulate the expression of MICA on the surface of human corneal epithelial cells (HCEC) that led to enhanced HCEC apoptosis *via* NKG2D presenting NK and CD8^+^ T cells (Wu et al., 2018). Furthermore, it has been shown that TNFα and IL-1β promote MICA expression on endothelial cells surface (Chaeuveau et al., 2014). Several key pathways are found to enhance MICA expression after cytokine treatment including the NFkB and mitogen-activated protein kinase pathways JNK, ERK1/2, and p38 (Fitau et al., 2006; Lin et al., 2012; Chaeuveau et al., 2014). Collectively these observations suggest that bystander activation and inflammation in tissues may both upregulate NK ligands on certain cell types and in certain circumstances although the mechanism for this is unknown at present.

## The Immunosurveillance of Senescent Cells in Tissues

**FIGURE 2:**
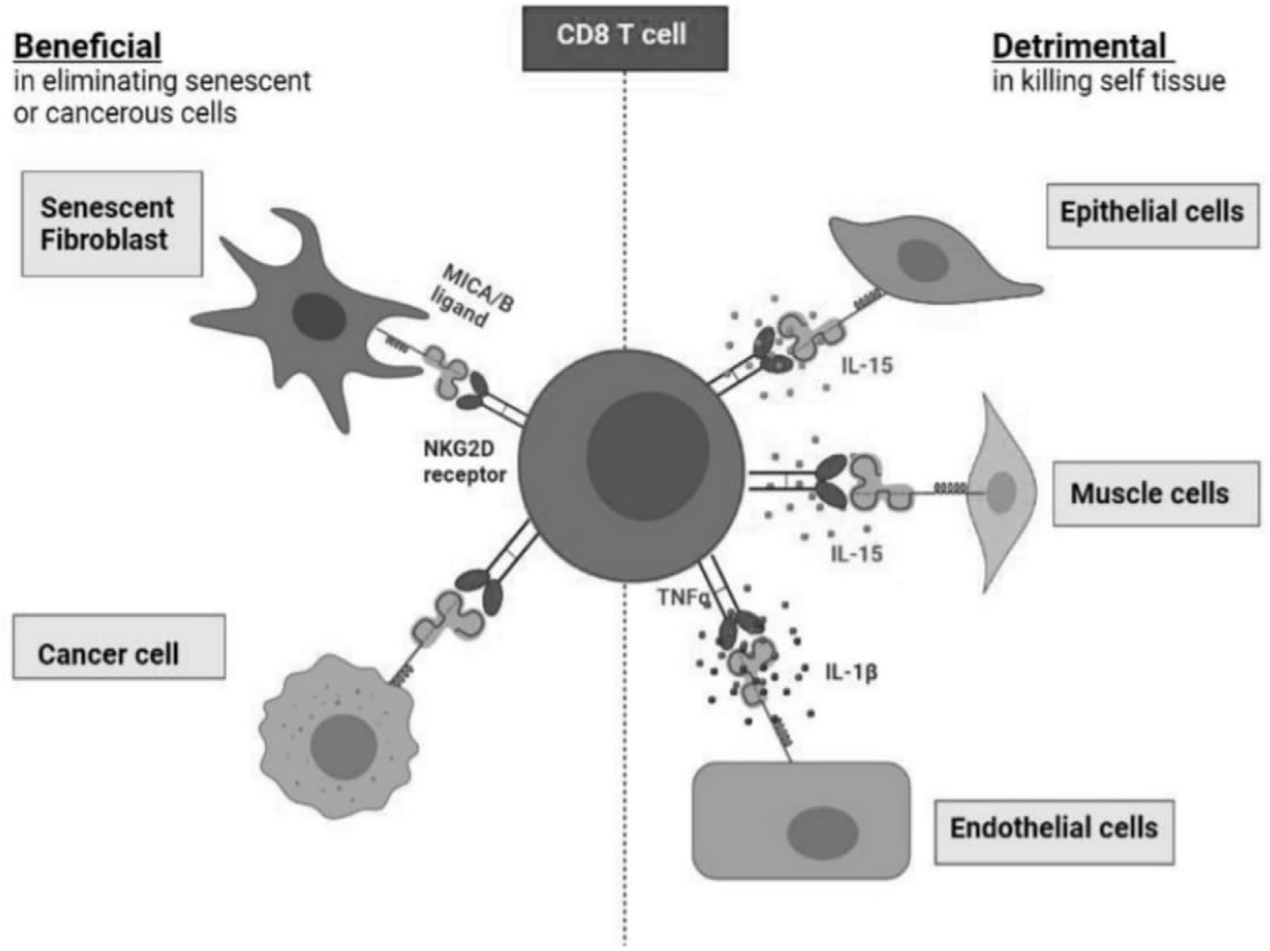
The benefits and detriments of NKR expression on healthy CD8^+^ T cells. Healthy CD8^+^ T cells expressing NKRs harness cytotoxic targeting of many cell types. NKR expression allows for the elimination of senescent fibroblasts as well as cancer cells which present the MICA/B ligands. However, NKR expression on CD8^+^. T cells can drive healthy tissue destruction *via* targeting of endothelial cells, muscle cells, and epithelial cells which also present MICA/B which is facilitated by various cytokines: IL-15, TNFα and IL-1β.

NK cells provide broad antigen independent protection against malignant and infected cells (Paul and Lal, 2017). It is likely that senescent T cells, especially within the CD8 compartment, may have a similar protective role (Pereira et al., 2020) (Figure 2). In addition to this, both NK cells and CD8^+^ T cells can also recognize and eliminate senescent non-lymphoid cells in tissues (Covre et al., 2020). Senescent non-lymphoid cells increase in all tissues during ageing and these populations can be recognised and eliminated by cells of the immune system, including senescent T cells (Covre et al., 2020). Different immune cell types including macrophages, neutrophils, natural killer (NK) cells, CD8^+^, and CD4^+^ T cells have been implicated in the surveillance of senescent tissue cells (Heonicke et al., 2012; Burton et al., 2018; Pereira et al., 2019).

Senescent cells become immunogenic by expressing stimulatory ligands like MICA/B that bind to NKG2D and activate NK cells killing (Gasser et al., 2005; Krizhanovsky et al., 2008; Sagiv et al., 2016). Moreover, by secreting cytokines, senescent cells can recruit immune cells into tissues and enable senescent cell clearance (Iannello et al., 2013). Functionally, we have shown that both NK and CD8^+^ T lymphocytes can target senescent cells through a NKG2D-dependent mechanism. Despite expressing high levels of NKG2D, the simultaneous presence of the inhibitory receptor NKG2A on senescent NK and CD8^+^ T cells may compromise their ability to eliminate senescent tissue cells. There is therefore a balance between activating and inhibitory receptor signalling on the same cell that has to be taken into consideration (Pereira et al., 2019). The expansion of NKG2A^+^ CD8^+^ T cells with age may explain why the immune system is less effective at eliminating senescent cells in old subjects, thus allowing them to accumulate compared to young individuals (Pereira et al., 2019).

However, NKR expression on CD8^+^ T cells can drive healthy tissue destruction *via* targeting of endothelial cells (Chauveau et al., 2014), muscle cells (Ruck et al., 2015), and epithelial cells (Zdrenghea et al., 2012; Wu et al., 2018) which also present MICA/B which is facilitated by various cytokines: IL-15, TNFα, IFN-y, and IL-1β.

## Senescent T Cells in Health and Disease

In this article we have discussed data suggesting that bystander activation by cytokines during immune responses can induce senescence in T cells that are unrelated to the infectious agent. This bystander activated (senescent) T cells are also induced to express NK receptors by these cytokines. The NK ligands on tissues can be themselves induced by inflammatory cytokines or are also constitutively expressed on senescent cell populations. While the senescent T cell population may have a broad protective role against tumours and infected cells, they may also be able to recognize and eliminate senescent cells from tissues. Previous studies indicate that the elimination of senescent tissue cells promotes health and alleviates age-related pathology (Baker et al., 2011). In these situations, bystander activation induced senescent T cells may promote health. However, in situations where there is extensive inflammation, these cells may have a destructive role because the cytokines would induce NK ligand expression on tissues. In this regard, persistent activation during the acute stage of leishmaniasis drives T cell senescence and homing of these cells to the skin. These senescent T cells secrete pro-inflammatory cytokines, exhibiting a senescence-associated secretory phenotype (SASP) that generates exacerbated inflammation. The exaggerated inflammation contributes to the progression of skin lesions and tissue destruction which has been shown in both mucosal leishmaniasis (ML) and cutaneous leishmaniasis (CL) patients (Bacellar et al., 2002; Antonelli et al., 2005; Crosby et al., 2014). The proportions of senescent T cells in the circulation and in the skin of these patients correlate with skin lesion size suggesting their contribution to the skin pathology that is observed.

More recently, infection with the SARS-CoV-2 virus that causes severe respiratory disease has led to a world-wide pandemic (coronavirus disease 2019, COVID-19; Merad et al., 2020). This infection mostly induces mild to moderate symptoms in younger individuals but induces devastating morbidity and mortality in some older individuals. A key hallmark of severe COVID-19 is exuberant inflammation in the respiratory tract of patients (Merad et al., 2020). As the proportion of senescent T cells with NK function increase during ageing and there is also extensive inflammation in tissues especially the lungs of the infected patients, the scene may be set where these T cells may recognize and destroy NK-ligand expressing cells in the lung epithelium and endothelial cells in this tissue (Akbar and Gilroy, 2020). A fundamental unanswered question is the nature of the trigger that may induce a potentially beneficial re- structuring of function in T cells during ageing and senescence to become highly destructive. An answer to this question would also be important for a wide range of other disease also where senescent T cells and extensive inflammation co-exist.
